# Eosinophilic esophagitis: published evidences for disease subtypes, indications for patient subpopulations, and how to translate patient observations to murine experimental models

**DOI:** 10.1186/s40413-016-0114-3

**Published:** 2016-07-15

**Authors:** Anne C. A. Mudde, Willem S. Lexmond, Richard S. Blumberg, Samuel Nurko, Edda Fiebiger

**Affiliations:** Department of Medicine, Harvard Medical School, and Division of Gastroenterology and Nutrition, Boston Children’s Hospital, 300 Longwood Avenue, Boston, MA 02115 USA; Division of Gastroenterology, Hepatology, and Endoscopy, Brigham and Women’s Hospital, Harvard Medical School, Boston, MA USA; Center for Motility and Functional Gastrointestinal Disorders, Boston, MA USA; Eosinophilic Gastrointestinal Disease Center, Boston Children’s Hospital, Boston, MA USA

## Abstract

Eosinophilic esophagitis (EoE) is a chronic inflammatory disorder of the esophagus and commonly classified as a Th2-type allergy. Major advances in our understanding of the EoE pathophysiology have recently been made, but clinicians struggle with highly unpredictable therapy responses indicative of phenotypic diversity within the patient population. Here, we summarize evidences for the existence of EoE subpopulations based on diverse inflammatory characteristics of the esophageal tissue in EoE. Additionally, clinical characteristics of EoE patients support the concept of disease subtypes. We conclude that clinical and experimental evidences indicate that EoE is an umbrella term for conditions that are unified by esophageal eosinophilia but that several disease subgroups with various inflammatory esophageal patterns and/or different clinical features exist. We further discuss strategies to study the pathophysiologic differences as observed in EoE patients in murine experimental EoE. Going forward, models of EoE that faithfully mimic EoE subentities as defined in humans will be essential because mechanistic studies on triggers which regulate the onset of diverse EoE subpopulations are not feasible in patients. Understanding how and why different EoE phenotypes develop will be a first and fundamental step to establish strategies that integrate individual variations of the EoE pathology into personalized therapy.

## Background

The large number of recent reviews on eosinophilic esophagitis (EoE) illustrates the importance of research on this disease and underlines the great interest that allergists, immunologists, and gastroenterologists have in this condition. Several of the world-leading experts, including (in alphabetical order) Drs. Aceves, Cianferoni, Dellon, Furuta, Liacouras, Oyoshi, Rothenberg, and Spergel, have recently provided detailed overviews on the state of the art in EoE research [1–12]. To avoid redundancy, we will only briefly summarize the diagnostic approaches to identify EoE and the general features of EoE pathology as we feel is necessary to facilitate the subsequent discussion on emerging evidences for the existence of EoE subpopulations.

EoE is classified as a primary eosinophilic gastrointestinal disorder (EGID) [13]. EGIDs constitute a heterogeneous group of diseases that, in addition to EoE, include eosinophilic gastritis, eosinophilic gastroenteritis, eosinophilic enteritis, and eosinophilic colitis. The unifying hallmark and diagnostic marker of all EGIDs is an eosinophil-rich inflammatory infiltrate of the affected mucosa. The etiology of EGIDs is generally not well understood. Tissue eosinophilia is typically considered to be of unknown origin, but the pathogenesis of all EGIDs appears to involve a complex interplay of genetic predisposition, exposure to food, and/or environmental allergens and Th2-type activation of the immune system [14, 15]. In this review, we focus on EoE because it is currently the most commonly diagnosed EGID [13], with a prevalence of 0.5–1 case/1000 persons and an incidence of approximately 1 new case per 10,000 persons per year [16], and is therefore the most extensively studied among the different varieties of EGIDs.

## Pathophysiological characteristics of EoE

### Diagnostic criteria

EoE is a clinicopathological disease. Quantification of esophageal tissue eosinophils combined with assessment of clinical symptomatology suggestive of esophageal dysfunction remains the gold standard in identifying patients with EoE [9, 17, 18]. The established histological standard for diagnosing EoE is the presence of esophagitis as characterized by >15 eosinophils per high power field in esophageal mucosal biopsies following adequate proton pump inhibitor therapy [17, 19]. EoE is defined as isolated to the esophagus, and all other recognized causes of esophageal eosinophilia must be ruled out prior to diagnosis. It is well acknowledged that this standard diagnostic approach by symptomatology and histology is not only an invasive diagnostic method, as esophageal biopsies are required, but also suffers from poor specificity [20, 21]. Esophageal eosinophilia can be found in other gastrointestinal (GI) disorders, and clinical symptoms associated with EoE are often non-specific, especially in young children [17, 19, 22–27].

In daily practice, distinguishing between EoE and reflux-associated eosinophil infiltration of the esophagus as seen in gastroesophageal reflux disease (GERD) remains particularly difficult, especially since the recent recognition of a new entity: PPI-responsive esophageal eosinophilia (PPI-REE). PPI-REE has similar clinicopathological characteristics to EoE but resolves following high-dose PPI therapy [7, 28–30]. GERD and EoE are not mutually exclusive, and the relationship between GERD, EoE, and PPI-REE is complex and not yet fully understood [31, 32]. In contrast to GERD, EoE pathology appears to be strongly allergen-driven. The comorbidity of allergic diseases ranges from 42 to 93 % in pediatric and 28–86 % in adult EoE patients, which is significantly higher than in GERD patients or the general population [30, 33–36].

Elevated serum IgE is found and used to identify allergen-sensitized EoE individuals that suffer from an IgE-mediated/allergen-driven disease phenotype. However, up to 50 % of EoE patients have normal serum IgE levels and, as defined by consensus using standard serum diagnostic approach, show no evidence for allergic sensitization in serum. In a conclusive opinion piece, it was recently argued that the pathogenesis of EoE is rather distinct from IgE-mediated food allergy [37]. The authors make the point that eosinophilic inflammation appears to be largely independent of IgE although food has been recognized as a trigger factor of EoE. Despite this recent review, common consensus is that, irrespective of the frequent absence of an allergic serum phenotype, EoE is classified as a Th2-type allergic disease because of its inflammatory esophageal mRNA pattern [19, 25, 38–41]. EoE was recently reviewed in detail from the perspective of the allergist [42]. How various allergic comorbidities potentially influence the EoE pathophysiology will be an important topic to study. The question as to whether EoE exists as an independent allergic/atopic disease or is most frequently found as a secondary manifestation in addition to another allergy needs to be addressed in large, ideally multicenter, cohort studies. Such studies will address important gaps in our understanding of the interplay of allergic conditions, which is commonly referred to as the atopic march. In this context, a recent publication demonstrated that EoE that develops in the context of skin sensitization has very unique features [43]. The type of EoE that develops after allergic sensitization at the skin appears to be critically mediated through the IL-33-ST2-basophil axis. This observation indicates that interference with this immune modulatory axis might provide another opportunity for individualized therapy of EoE provided that the finding can be confirmed in other human EoE cohorts.

### Treatment strategies

Clinicians treat EoE to reduce, or ideally resolve, esophageal inflammation and to resolve symptoms. The most commonly used therapeutic strategies for EoE are proton-pump inhibitors, which are also important in the diagnostic phase; topical corticosteroids, most often in the form of swallowed fluticasone or liquid budesonide; and/or dietary interventions. Several dietary strategies are used, such as the six-food elimination diet, targeted food elimination based off allergy testing, and elemental diet therapy. Dilatation of esophageal strictures is also a treatment strategy used to relieve EoE associated symptoms, such as dysphagia; however, it has no effect on inflammation [9]. The reported response rates to either dietary interventions or topical steroids vary greatly from 50 % to >90 %, with the most often reported response rate for commonly used dietary interventions and local steroids being around 70 %. The highest response rates are achieved by treatment with elemental diets; however, this therapy is associated with high costs and low compliance due to unpleasant taste [1, 9, 18, 22, 27, 44–50]. Even though there are studies that have shown benefits from dietary therapy in adults, the therapeutic success of dietary intervention for adult EoE is still debated amongst experts [51].

### Evidences for EoE subpopulations

Generally, it is important to stress that the concept of differential disease subphenotypes and subgroups is not new to allergy research. For asthma, the most common allergic disease, disease subtypes and individualized therapy have, in fact, already been integrated in clinical practice and are known to be beneficial in guiding therapy [52–54]. Basic research strategies have also been established which model different asthma phenotypes and mimic individual patient subpopulations [55]. Little attention has been paid to EoE subtypes in clinical practice so far, and basic research on EoE is only starting to integrate the idea of EoE subtypes into experimental strategies to understand EoE pathophysiology.

### EoE patients cluster in subpopulations based on age and clinical characteristics

Clinical presentation of EoE is highly age dependent. Infants tend to suffer from feeding difficulties and failure to thrive. Children typically experience abdominal pain, nausea, vomiting, and heartburn, whereas adolescents and adults tend to present with dysphagia and episodes of food impaction [1, 56]. Recently, new manuscripts describing the adult onset of EoE have been published [26, 57, 58]. The comparative analysis of the roles of IgE and IgG4 in adult EoE showed elevated serum levels of IgG4 with food-specificity in adult EoE patients and a 45-fold increase in IgG4 in homogenates of esophageal specimens from adult EoE patients over controls. In support of the IgE-independence of the adult EoE, no effect of omalizumab, the most commonly used IgE-blocking therapy, was observed [57]. Combined, these results indicate that adult EoE might be IgG4-mediated rather than an IgE-mediated allergy and, therefore, an EoE subpopulation in itself. It is, of course, equally possible that the interplay between antigen-specific IgG4 and IgE signals differentially shapes adult EoE on the cellular level. The topic of IgG4 and IgE in fuelling human allergic diseases was recently discussed in detail and the nature of EoE as a ‘modified Th2-type’ disease was put forward [59]. However, mechanistic explanations remain speculative in the absence of experimental data on the topic.

Proton pump inhibitor-responsive esophageal eosinophilia (PPI-REE) has recently been described [60] and may represent another subtype of EoE. PPI-REE has similar clinicopathological features to EoE, is also an allergic disease, and presents with Th2-associated characteristics comparable to EoE. Furthermore, the molecular transcriptome of PPI-REE and EoE show significant overlap. Current consensus suggests that PPI-REE and EoE have a common pathogenesis and that PPI-REE might constitute a subgroup of EoE [29, 61].

Another recently described phenotype of EoE consists of EoE patients with an extreme narrow-caliber esophagus. This patient group is characterized by a longer symptom duration and a disease phenotype that is rather refractory to steroid treatment [61].

### EoE patients and comorbidities

EoE is more prevalent in patients suffering from other disorders. For example, an association between EoE and celiac disease has been suggested. However, contradicting reports are found in the literature, and no mechanistic studies have been performed [62–64]. Additionally, several case reports and case series found a higher prevalence of EoE in children with esophageal atresia [65–68]. Microdeletions encompassing the Forkhead box (FOX) transcription factor gene cluster have been shown to be associated with esophageal atresia. Mouse studies have shown that binding sites for Foxf1, one of the encoded proteins of the FOX gene cluster, include the promoter region of genes for inflammation, such as eotaxin. The expression of eotaxin-3 is known to be increased in patients with EoE. In combination, these results led to the hypothesis that mutations in the FOX gene cluster play a role in the development of esophageal atresia and EoE [64, 69]. Finally, an elevated prevalence of EoE in patients with inherited connective tissue diseases (CTDs), particularly those associated with hypermobility, has been described [7, 64, 70]. It has been proposed that these patients represent a subpopulation, referred to as EoE-CTD, with specific clinical characteristics such as lower BMI and an elevated risk of extra-esophageal eosinophilic gastrointestinal disease. Increased TGF-β signaling has been proposed as a molecular connection between EoE and CTDs [70]. Additionally, association of EoE has been reported with celiac disease in the pediatric population and celiac patients with active disease [62, 71].

### EoE patients cluster in subpopulations based on their esophageal inflammatory mRNA pattern

Esophageal tissue inflammation is described to be highly variable within the EoE patient group [7, 24]. Our research group recently confirmed this observation with multiplexed mRNA pattern analysis on esophageal biopsies [72] and classical mRNA pattern studies with qRT-PCR [73, 74]. Importantly, we discovered that EoE patients cluster in subgroups based on their mRNA expression profile, publishing three research articles in which we described EoE subpopulations with distinct inflammatory tissue characteristics [72–74]. The first group, which we here refer to as LTC4S-EoE, is characterized by a more pronounced esophageal Th2-type inflammatory profile including TSLP and is typified by high expression of leukotriene C4 synthase (LTC4S, [74]). Leukotrienes are known to actively contribute to allergic inflammation. Interestingly, esophageal levels of cysteinyl leukotrienes have been described not to differentiate EoE patients from controls [17, 38], and the literature on the effect of leukotriene receptor antagonist therapy in EoE is inconclusive. Several case series ascribe therapeutic benefits to the application of leukotriene receptor antagonists in EoE patients [75–77]. On the other hand, leukotriene receptor antagonists have also been reported to be ineffective in EoE [78] or only effective in a patient subset [79]. At this point, it is tempting to speculate that the definition of an EoE patient subpopulation that is defined by LTC4S mRNA levels might be a first step to identify individuals that benefit most from treatment with leukotriene receptor antagonists.

Another EoE subgroup is defined by low transcript levels of LTC4S but elevated IL23 transcripts (referred to as IL23-EoE, [74]). IL23 is considered to be associated with Th17 immune responses [80], such as in a severe type of asthma [81–83]. The IL23-EoE phenotype might thus have a more pronounced Th17 phenotype. The latter finding would be in line with the concept that EoE can be a manifestation of asthma in the esophagus [84]. Defining such a subgroup might have significant consequences on patient management since asthma treatment strategies could be considered.

In an independent study, we described another EoE subtype that is associated with an increased CD1d-restricted, invariant chain natural killer T (iNKT) cell compartment (referred to as iNKT-EoE, [73]). Jyonouchi et al. also discusses the role of iNKT cells in EoE, explaining that an iNKT cell-mediated Th2-type cytokine response can lead to allergic inflammation in EoE [85]. Our study found iNKT-EoE to be an early-onset form of EoE, predominantly present in children under six years of age. Interestingly, LTC4S-EoE and iNKT-EoE have overlapping features as these subgroups appeared to present with more food allergy and higher levels of serum IgE. Further subgroup analysis implied that iNKT-EoE patients fall rather uniformly into the LTC4S-EoE group, as these patients also express high levels of LTC4S, suggesting that iNKT-EoE might be a subentity of LTC4S-EoE. This topic deserves integration into the growing discussions about EoE and classical food allergy [86].

The distribution analysis of mRNA expression levels of the mast cell marker carboxypeptidase3 [72, 74] also supports the existence of a subpopulation of MC-EoE patients. Rothenberg and colleagues recently published that in EoE patients, mast cells are associated with dysphagia [87]. In experimental murine EoE, mast cells have been shown to play a role in muscular cell hyperplasia [88]. In addition, it has been suggested that mast cells modulate esophageal contraction by increasing smooth muscle contractility, which could underlie dysphagia [89]. In summary, the precise role of mast cells in EoE warrants further investigation.

### Experimental murine eosinophilic esophagitis

Defining clinical subtypes in EoE patients is a first essential step towards establishing novel treatment strategies that take individual variations of the EoE pathology into account. Going forward, human cohorts might be less useful here because mechanistic studies are hardly feasible in patients.

One perfect example for the problem of doing functional studies that define cause and effect in EoE pathology are studies on the role of the microbial signals for EoE development. Such studies are highly important in view of the recent description of the EoE specific microbiome [90] and the rapidly growing knowledge of the role of microbial signals in allergies of the GI tract [91, 92]. Germ-free humans do not exist and, even in mice, microbiome studies are challenging and highly costly. Irrespectively, the use of germ-free murine models provides us with unique opportunities to study the impact of individual microbial strains on the pathology of EoE.

### Commonly used strategies to model EoE in mice

Experimental strategies to understand pathophysiologic differences of EoE as seen in patients require murine disease models that phenocopy human EoE and its subtypes in vivo as closely as possible. Using a variety of allergens, diverse types of sensitization, and different types of challenges, several murine models of experimental EoE have been described in the literature. For a conclusive review on the established experimental models of EoE, we refer to Mishra et al [93].

The most commonly used experimental EoE model uses intranasal application of *Aspergillus* in a sensitization/challenge type of model [93, 94]. The esophageal infiltrate in this model contains mast cells. Therefore, the *Aspergillus* model might be well suited to study MC-EoE. Since *Aspergillus* is also a common antigen in asthma, this strategy could also be useful for studying subtypes of EoE that are associated with asthma. In contrast, the combination of peanut antigen and sensitization with aluminum hydroxide as adjuvant (PA/alum) has been established for inducing iNKT cell infiltration in EoE [93, 94]. It is therefore likely that the latter model is best suited to study iNKT-EoE. Presently, no reports on age-dependency of murine EoE models are found in the literature, whereas age-dependency and a role of the microbial signals early in life have been demonstrated for the development of allergic asthma [95].

### Using humanized mice to study mucosal allergy – implications for eosinophilic esophagitis

**Fig. 1:**
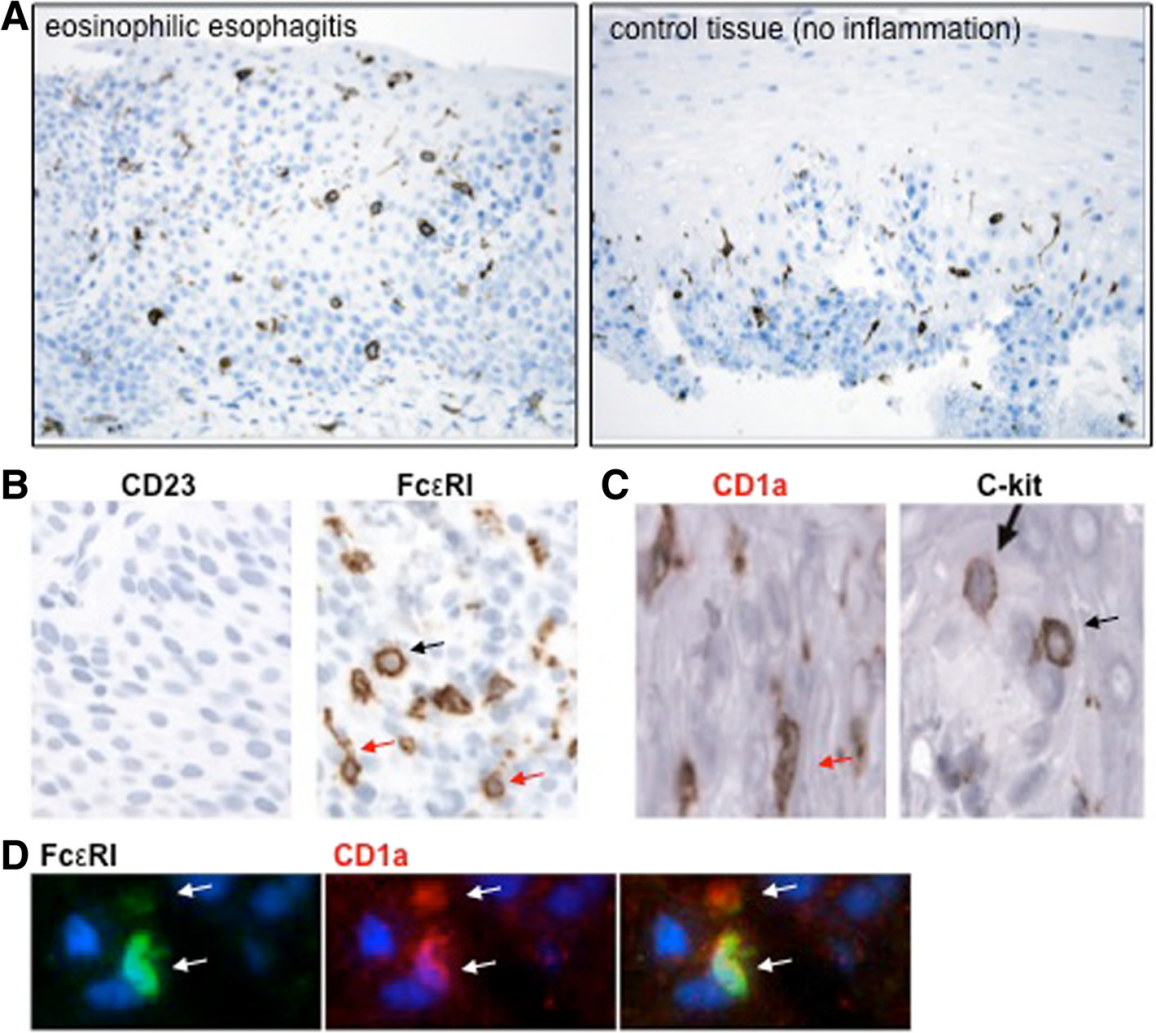
IgE Fc receptor expression of the human esophagus. **a** Dendritic cells of the esophagus express FcεRI. Anti-FcεRIalpha staining in esophageal tissue. Left: EoE, right non-inflamed control tissue. **b** Comparative expression analysis of CD23 and FcεRI in EoE tissue. **c** Esophageal dendritic cells (CD1a staining) and esophageal mast cells (C-kit staining). **d** FcεRI/CD1a immune fluorescence histochemistry of the esophagus. Finding originally published in [97, 99, 100]

Following the established concept that EoE is, at least in a considerable number of patients, a manifestation of an IgE-mediated allergy, one has to consider that experimental murine EoE needs to be studied in models that faithfully mimic the human IgE network [96]. Importantly, a direct comparison of the murine and human IgE Fc receptor expression profile during the in situ characterization of IgE-binding structures of the human esophagus [97] revealed a substantial shortcoming of murine models of EoE. We found that FcεRI, the high affinity IgE Fc receptor [96, 98], was the only IgE receptor that was robustly expressed in the esophageal tissue of patients with EoE (Fig. 1 and [97]). Dendritic cells (DCs) and mast cells were the predominant FcεRI expressing cells, while eosinophils hardly expressed FcεRI in the esophagus of EoE patients [97]. Comparative mRNA pattern analysis showed that more DCs and, therefore, also more DC-bound IgE is found in the non-inflamed esophagus than in EoE [72, 73, 97]. Additionally, we found that FcεRI on DCs critically contributes to the cell-bound fraction of the human IgE pool in peripheral blood of EoE patients [97, 99, 100]. In mice, however, DCs do not constitutively express FcεRI and, therefore, cannot form a DC-bound IgE pool in tissue or blood [101, 102]. For a summary of the constitutive expression of FcεRI in humans, see Table 1. In conclusion, it is fair to postulate that murine EoE models fail to faithfully represent the DC-specific fraction of the cell-bound IgE pool as found in EoE patients.

**Table 1:** Cellular expression profiles of FcεRI isoforms in humans and wild type mice. Composition of the tetrameric isoform: IgE binding alpha chain, beta and the gamma dimer. Alpha chain contains the IgE binding site. Beta and gamma are the signal transducing subunits. Composition of the tetrameric isoform: IgE binding alpha chain and the gamma dimer. For review see also [96, 107]. Note that only a minor fraction of eosinophils in esophageal tissue of EoE patients expresses FcεRI [97]

	Human FcεRI	Murine FcεRI
Cell type		
Mast cell	αβγ2	αβγ2
Basophils	αβγ2	αβγ2
Dendritic cells	αγ2	--
Eosinophils in tissue	αγ2	--

As corroborated by studies from our own laboratory, allergic Th2-type diseases such as EoE are on the list of human conditions that are not fully modeled by the murine immune system in their complexity [103]. For many years, the inconvenient reality of divergences in human and murine immune responses has prompted investigators to dedicate active research effort to the humanization of mice as summarized and discussed recently by Grisham and colleagues [104]. The use of humanized models in which immune compromised animals are repopulated with human cells has proven problematic for allergy research because such animals do not mount proper IgE responses [104, 105]. Our research group has recently worked on a humanized model in which animals that lack murine major histocompatibility complex class II (MHC II) and instead express human leukocyte antigen DR1 (HLA-DR1) were studied [106]. Importantly, such humanized mice presented with robust serum IgE levels indicative of an improved allergy model [106]. However, the DC-bound IgE pool was not reconstituted in these animals, which renders this model inappropriate for research questions that address the role of IgE in EoE (Fiebiger lab, unpublished observation). As an alternative strategy, our laboratory has established mouse strains in which a transgenic (TG) approach was chosen for the expression of the IgE-binding alpha chain using the DC-specific CD11c promoter. DCs of such mice (referred to as IgE_R_-TG mice [101, 107–109]) express FcεRI and are IgE-loaded like their human counterparts [101, 102, 107, 108, 110]. It is important to stress that several of the proposed IgE_R_-TG strains are characterized in detail and have been published by our group as well as others.

Research applying IgE_R_-TG mice has provided surprising insights into the physiological role of FcεRI-bound IgE in allergy. None of the IgE_R_-TG strains developed a spontaneous allergic phenotype, implying that the presence of cell-bound IgE on DCs does not drive the development of allergies, including EoE. Using three independently generated IgE_R_-TG strains on two different backgrounds, less severe Th2-type allergic inflammation was found in the TG animals than in their wild-type counterparts, which did not have DC-bound IgE. Experiments with human DCs confirmed that IgE-crosslinking dampens the TLR-ligand-induced production of Th2-promoting cytokines, validating that experiments in the IgE_R_-TG mice yield results that are directly relevant for human allergy [107]. Furthermore, FcεRI on human monocytes and DCs has been shown to contribute to IgE clearance [111], substantiating that FcεRI on DCs is regulatory [101, 102]. How immune regulatory IgE-mediated signals operate and/or fail in EoE is currently unknown, and the role of DC-bound IgE in the pathophysiology of EoE has not been addressed experimentally as of yet. Therefore, we propose that IgE_R_-TG mice should be used as an improved model for studying mechanisms that regulate allergic IgE-mediated inflammation in EoE and the development of EoE disease subtypes.

## Conclusions and future perspectives

Cumulative evidences support the existence of subtypes of EoE. Inflammatory tissue mRNA pattern analysis might be a feasible approach to define certain EoE patient subpopulations, but more studies are required to validate the approach. Currently, the development of experimental strategies that aim at providing mechanistic insights into cellular and molecular pathways that regulate the development of EoE subtypes are highly important. New insights into mechanisms responsible for the manifestation of diverse pathologies are a prerequisite to establish the concept of different EoE subtypes more convincingly. Research efforts along this line will lead to improved understanding of causative triggers for EoE, in general, and more specifically of factors that define individual EoE subtypes. A better understanding of diverse EoE pathologies, in turn, has the potential to direct the development of new therapeutic approaches with a focus on individualized patient therapy.

## Abbreviations

CTDs, connective tissue diseases; DCs, dendritic cells; EGID, eosinophilic gastrointestinal disorder; EoE, eosinophilic esophagitis; FcεRI, high affinity IgE Fc receptor; GERD, gastroesophageal reflux disease; IgE_R_-TG, FcεRI transgenic; iNKT, invariant natural killer T cell; MC, mast cells; PPI-REE, PPI-responsive esophageal eosinophilia
